# Perceptions of Nature and Access to Green Space in Four Urban Neighborhoods

**DOI:** 10.3390/ijerph16132313

**Published:** 2019-06-29

**Authors:** Justine S. Sefcik, Michelle C. Kondo, Heather Klusaritz, Elisa Sarantschin, Sara Solomon, Abbey Roepke, Eugenia C. South, Sara F. Jacoby

**Affiliations:** 1 School of Nursing, the University of Pennsylvania, Claire M. Fagin Hall, 418 Curie Blvd, Philadelphia, PA 19104, USA; 2 Northern Research Station, USDA Forest Service, 100 N. 20th St, Ste 205, Philadelphia, PA 19103, USA; 3 Department of Family Medicine and Community Health, the Perelman School of Medicine, the University of Pennsylvania, 3737 Market St 9th Fl., Philadelphia, PA 19104, USA; 4 NaturePHL Program, Department of Education, Schuylkill Center for Environmental Education, 8480 Hagys Mill Rd, Philadelphia, PA 19128, USA; 5 Penn Injury Science Center, Perelman School of Medicine, the University of Pennsylvania, 423 Guardian Drive, Philadelphia, PA 19104, USA; 6 Center for Public Health Initiatives, Perelman School of Medicine, University of Pennsylvania, 3620 Hamilton Walk, Anatomy Chemistry Rm 141, Philadelphia, PA 19104, USA; 7 Department of Emergency Medicine, Perelman School of Medicine, the University of Pennsylvania, Blockley Hall 408, 423 Guardian Dr, Philadelphia, PA 19104, USA; 8 Department of Family and Community Health, School of Nursing, the University of Pennsylvania, Claire M. Fagin Hall, room 412, 418 Curie Blvd, Philadelphia, PA 19104, USA

**Keywords:** green space, nature, focus groups, low-resource neighborhoods

## Abstract

Health benefits have been linked to spending time outdoors in nature and green space. However, there is some evidence of inequities to accessing safe outdoor space, particularly in low-resource communities. The primary aim of this study is to assess attitudes towards nature and use of green space in urban areas. A secondary aim is to describe perceptions of physician-initiated nature prescriptions that target local pediatric populations. We conducted six focus group interviews with 42 residents who were guardians or caretakers of children living in low-resource neighborhoods in Philadelphia, PA. We analyzed interview data using a conventional content analysis approach. Three major themes emerged: (1) perceived benefits of being in nature (physical and mental health benefits), (2) barriers to time spent in nature (unsafe and undesirable conditions of local parks), and (3) desired features of outdoor green spaces (amenities that would increase park use). Additionally, we describe participants’ reactions to the idea of a pediatrician-delivered prescription for outdoor green space exposure for a child in their care. Adherence to nature prescriptions programs may hinge on local green space resources, as well as experiential and perceptual barriers and facilitators to nature and park accessibility among caregivers tasked with fulfilling a nature prescription for a child in their care.

## 1. Introduction

A growing body of evidence suggests that spending time in nature can benefit human health [1,2,3,4]. Living in greener environments often results in higher levels of physical activity [5,6]. Green space exposure may also prevent or mitigate stress, anxiety, and depression [7,8,9] in both children and adults [10], especially in urban environments [11,12,13,14,15,16,17,18,19] where green space offers respite and opportunity for interactions among neighbors in ways that promote social cohesion and collective efficacy [11,20,21,22]. Exposure to natural environments can also minimize symptomatology associated with common pediatric health conditions, for example, it has been shown to improve attention [23,24] and lessen symptoms in children diagnosed with attention deficit hyperactivity disorder (ADHD) [16,25,26]. 

The President of the American Academy of Pediatrics, Kyle Yasuda, declared that connecting children and families with nature was as one of the Academy’s main priorities for 2019 [27]. Despite the myriad potential health benefits of exposure to nature and green spaces, many children spend little to no time outside on a regular basis [28,29,30,31]. In addition, access to parks in urban areas is not guaranteed, and existing urban parks are often underutilized [32,33]. Das et al. found that race and foreign-born status were associated with park underutilization in Minneapolis, Minnesota [34]. 

There is a growing evidence base informing our understanding of the inequities in park access and utilization [35,36,37,38]. Safety concerns are often a strong determinant for lower park use in low-resource neighborhoods. Parks can provide public, un-surveilled space and thus may become settings where social disorder and crime are more common [39]. Previous research has demonstrated that crime densities are higher in Philadelphia parks when compared to a random selection of street intersections across the city [39]. Gun violence in parks, in particular, has been shown to have a long-lasting negative impact on park use [40]. Other concerns include motor vehicle traffic, lack of lighting, and poor maintenance [39,41].

On the other hand, a study of 48 parks in low-income neighborhoods of Los Angeles found that while the presence of homeless populations and drug users deterred park utilization to some degree, the presence of organized programs and activities was a stronger predictor of park use [42]. Park amenities including staffing, programs, events, maintained landscaping, and infrastructure and facilities might draw more users into urban green spaces [43,44]. 

Park access, especially in low income neighborhoods, may play an important role in community health. Research on exposure to or use of parks (as opposed to urban “nature” or “green space” in general) has found a positive association with community social relationships, including collective efficacy [45,46]. Parks have been shown to engender mental health benefits [47], which could be, in part, due to improved social interactions, increased opportunities for physical activity, or lower stressful exposures [48]. One randomized, controlled trial of greening interventions to park-like spaces (open lots) throughout Philadelphia, PA found that residents’ feelings of depression and worthlessness significantly decreased when exposed to lots that had been transformed into greened spaces compared to control lots where no intervention had occurred [49].

The evidence demonstrating health benefits from time spent in green spaces, parks, and natural environments has led to nature-prescription programs that have emerged as preventative and curative health interventions. Over 100 programs exist in which healthcare providers, predominantly pediatricians, give a “prescription for nature” to their patients [50]. These programs vary widely in their content, execution, and target populations. For example, some programs provide counseling about nature and text message reminders [17], while others do targeted engagement with recreation systems through referrals or program coordination [51]. 

Much remains unknown about the most effective way to structure these kinds of nature prescriptions and programs so that patients will be successful in following through. Unrecognized barriers to access and interest in these programs, if not addressed, will prevent adherence and effectiveness [52,53]. Prior work has examined behaviors and perceptions associated with park use [34,42,44]. Only one prior study has explored perceptions of parks with the purpose of informing park prescriptions [54]. Uijdewilligen et al. [54] concluded, based on a series of surveys and focus groups with residents of northern Singapore, that parks prescriptions would need to help patients plan for outdoor physical activity, build social support, and improve proximity of accessibility to parks and park safety. 

We present findings from focus group interviews with guardians and caretakers of children from four low-resource neighborhoods in Philadelphia, PA. The primary aim of this study is to assess attitudes towards nature and use of green space in urban areas. A secondary aim is to describe participants’ perceptions of physician-initiated nature prescriptions that target local pediatric populations. These results are particularly relevant for healthcare providers who serve communities with limited public resources and integrate nature prescriptions in their approach to chronic disease prevention and management.

## 2. Materials and Methods 

### 2.1. Design

This study used a qualitative descriptive design, in which focus group interviews were the primary data collection strategy [55,56]. This design was selected to elicit in-depth insights from participants on their perspectives towards accessing green space in their neighborhoods and generate descriptive summaries. Focus groups interviews were chosen so that data could include comparative insights and a greater range of ideas than may be possible through individual interviews [57]. Limited demographic information on each focus group participant was obtained through a brief pre-focus group survey.

### 2.2. Recruitment

**Figure 1:**
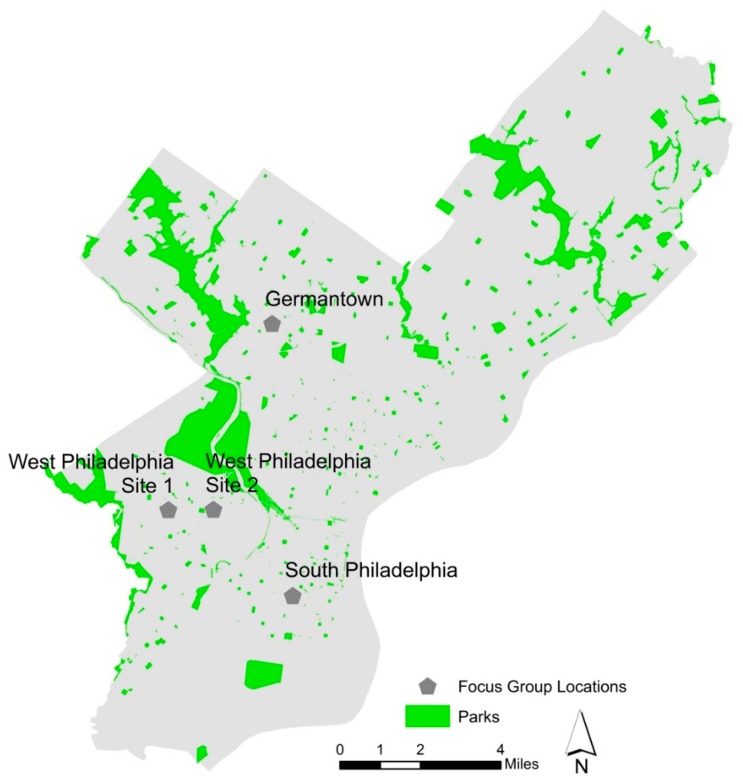
Focus group locations compared to the location of parks in Philadelphia, PA.

A convenience sample of guardians and caretakers of children was recruited through fliers posted in four urban neighborhoods surrounding Children’s Hospital of Philadelphia (CHOP) outpatient clinics where a nature prescription program would soon be implemented (Figure 1). Fliers were displayed at community meetings, Early Head Start centers, elementary school parent-teacher association (PTA) meetings, libraries, places of worship, and Women, Infants and Children (WIC) offices. Inclusion criteria for the focus group participation were proficiency in spoken English, being 18 years or older, and residence or employment within one of the neighborhoods surrounding a clinic.

### 2.3. Procedures

The University of Pennsylvania’s Institutional Review Board approved this study. Six focus groups were conducted at private rooms in community centers and places of worship near each clinic. Written informed consent and demographic information was obtained prior to each focus group. Focus group interviews were facilitated by two experienced moderators guided by a semi-structured interview guide that was developed by the research team. The focus groups averaged 60 minutes in length, and participants received a $10 gift card incentive for their time.

### 2.4. Coding and Data Analysis

Audio recordings of the focus groups were transcribed by a professional transcriptionist and then reviewed against original audio files for accuracy. Transcripts were then uploaded into Atlas.ti Version 7 (Scientific Software Development GmbH, Berlin, Germany) to store, code, and manage the data. Interview data were analyzed using conventional content analysis, which involved reading and re-reading focus group transcripts, coding small meaning units (first-level codes), developing a codebook, sorting the codes based on their relationships to other codes to create categories, coding larger text based on the categories, and developing themes [58,59]. The lead author (J.S.S.) completed all first level coding. From the first level coding, the research team developed categories and the codebook. J.S.S. and a research assistant then coded two transcripts independently using the codebook, after which the codebook and core definitional criteria were discussed and reconciled until there was 100% agreement. J.S.S. then completed coding of the remaining transcripts and the team determined final themes.

### 2.5. Trustworthiness

Several approaches were taken to ensure trustworthiness of the analysis [60]. First, an audit trail was kept to outline the process and decision points that guided the data analysis. This included a detailed description of initial codes, categories, and final themes, and the rationale for analytical decisions made throughout the study. Second, we triangulated our findings by engaging an interdisciplinary research team in review of the data, codes, categories, and themes to establish consensus on the analytical decisions and overall study findings.

## 3. Results

### 3.1. First Described are the Participant and Neighborhood Characteristics Followed by a Presentation of the Three Themes that Emerged and Findings Related to Perceptions of Physician Generated Nature Prescriptions

#### 3.1.1. Participant and Neighborhood Characteristics

Forty-two individuals participated in one of six focus group interviews (ranging from 4 to 13 participants). The majority of participants identified as female and Black (Table 1). Socioeconomic characteristics of the four study neighborhoods are shown in Table 2. Three of the neighborhoods were predominantly comprised of Black/African American residents, and one predominately comprised of Asian and White residents. Twenty-five to 44% of residents in these neighborhoods lived in single-parent households with a median household income between US $20,840 and $42,903. 

All four neighborhoods contain small “pocket” parks, although significant variability existed in neighborhoods’ density of and proximity to parks (see Figure 1). 

#### 3.1.2. Access to Information

Most participants reported that information about upcoming events and programs in local green spaces was easily accessible through a mixture of internet-based resources and public postings in their community (see Table 3).

### 3.2. Themes

Analysis revealed the following three themes: perceived benefits of being in nature, barriers spent to time in nature, and desired features of outdoor spaces.

#### 3.2.1. Perceived Benefits of Being in Nature

A perceived benefit of being in nature was the ability to be involved in a range of activities and engage in exercise. Activities ranged from attending events in local parks (e.g., concerts, movies, and baseball games) to engaging in different forms activity per preference (e.g., biking, climbing, fishing, and skating).

One parent described how she encouraged her children to be outside and increase physical activity. She specifically wanted to increase her son’s exercise time to decrease his weight to manage his obesity and diabetes. Some parents and grandparents shared that they bring their children outside to parks and playgrounds to facilitate play and expend energy. Some brought their children to park camps, which were primarily outdoors, and offered physical activities such as basketball, hiking, and swimming. A participant who worked with an after-school program that brought children to an outdoor recreation area with playgrounds, pools, and wooded areas for hiking remarked, “It is something kind of transformative that happens when they’re out in that type of nature”, referring to their increased playfulness. 

Some participants routinely enjoyed spending time in nature. For example, one mother shared her outdoor routine with her children, which did not cost her money:

Being a mom of little ones, I spend at least four days a week in the park. I actually enjoy it…We could be out riding our bikes. I love picnics…The kids love the geese. We’ll take some pieces of bread. So I enjoy the outdoor life. I enjoy the park life. And it’s free, so I love it.

Participants also reported improved mental health as a primary benefit of spending time outdoors. Specific mental health improvements included being able to think more clearly, feeling rejuvenated, and achieving a refreshed state of mind. One participant described his experience of going to a large park as “…a place of refuge for me in a sense of when I leave, I feel like a totally different person for a few days.”

#### 3.2.2. Barriers to Time Spent in Nature

Several barriers to time spent in nature were discovered (see Table 4). Lack of safety in local parks and playgrounds was the most commonly cited barrier to spending time in nature. One participant stated, “I got one word for these parks around here. They are infested.” Another participant reflected:

“I know this is illegal, but there should be a park if you just want to do illegal stuff. For all of you who want to do illegal activity, this is your park. But the rest of us who just want to play cards or play checkers, or just want to have clean fun, this is ours.”

For some participants, the perception of crime and danger in local outdoor green spaces was dependent on the time of day. One mother shared, “I keep my son outside as much as possible. Before the sun even go(es) down, he’s in the house. Because there’s too much activities going on out here in our area.” 

Other examples of barriers to spending time outside included dislike of the natural environment, outdoor conditions, and the physical conditions of local parks (Table 4). One participant stated that the conditions of local parks compelled her to travel outside her neighborhood to spend time with her children outdoors: “…I don’t always want to drive to get away. But if I don’t want to be picking up trash and drug paraphernalia and all kinds of stuff, then I have to go where…somebody’s cleaning the park’’.

A few participants felt that the fact that parks in their neighborhoods were unsafe and unmaintained demonstrated larger racial and economic biases in allocation of city resources. These participants perceived that parks and public green spaces in predominantly white neighborhoods were kept safer and more desirable. One participant also perceived a relationship between park improvement and increasing gentrification pressures in her neighborhood:

“That park was grimy for years. But with gentrification, then they want to fix it up. I’m like why didn’t you fix it up when my son was 3? Now you wait until…you’re trying to attract more Caucasians.”

Other participants expressed frustrations with the condition of their local parks and that “…you shouldn’t have to leave your immediate neighborhood to go someplace else.” In one focus group, participants discussed the disproportionate allocation of the city’s tax revenues for park investment. One participant shared her perception that

“… [the city] can spend a lot of money and taxes in one section, and then in another section you’re only spending $ 1000 or $ 500–$ 800 in taxes. And it’s just not the same. And that’s the messed [up] part. It’s basically saying well, if you’re poor, then you don’t get to have a safe park. But if you can afford to pay this extra, then we’ll look out for you.”

Some participants said that they did not have the option to travel to nicer parks further from home. In most cases this was due to financial hardship or health limitations. One mother stated that she does not always have a working car and she was worried that her autistic son might have a “melt down” on the bus or during a long car drive when she does have access to a car. Another mother reported that she and her children spent most of their time inside the home because she did not want to use up gas in her car to bring her child to more distant park that was safer than her neighborhood.

#### 3.2.3. Desired Features of Outdoor Green Spaces

Participants voiced desires for local outdoor spaces that were clean, well maintained, and safe. In the absence of these attributes locally, some people traveled out of their local communities to “nicer areas” to spend time outdoors. One participant shared

“For me, my reason for driving the longer distance as opposed to staying local, it’s for number one, safety reasons. Number two, it’s cleaner. The atmosphere is better. Safety, the atmosphere is cleaner, the beauty of going far out. You would never catch me at some more local parks.”

In addition, participants described other outdoor features that would be desirable in local green spaces, including playgrounds and jungle gyms surrounded by gates for children’s safety, autism-friendly parks, and outdoor pools. Regular police patrol and surveillance cameras were also described as desirable features to increase safety. 

Finally, access to structured and educational outdoor activities were also desired features. One participant reflected that this is particularly important for younger community members, stating

“…a lot of people say to get outside more, especially to the young folks in our community, but there’s not technically a lot of things for us to do outside. So give us some activities…”

Ideas generated included giveaways such as free Frisbees and passes for bike rentals which could provide youth with more structured play.

### 3.3. Perceptions of Physician Generated Nature Prescriptions

Each of the focus groups concluded with a discussion about a hypothetical scenario in which participants were asked to imagine that they brought a child in their care to a pediatrician who prescribed time outdoors in green space (a nature prescription) as a health intervention. There was a mix of negative and positive responses to this scenario. 

A few participants were highly receptive to the idea of a nature prescription for their children. One mother expressed

“If my pediatrician recommended that we spend more time outdoors, my response would be like ‘Wow, that’s interesting. Because we was planning to go to the park when we left here.’ So I would be excited to know that what I’ve been doing all along actually helps or should be done. And I would ask him, ‘Do you have any – a list of parks to share that I haven’t been to yet?’...I would be really enthused at the fact that what I’ve been doing all along is what needs to be done.”

However, most participants questioned their ability to follow-through with the recommendation. Lack of safety in local green spaces was a major barrier. One mother hypothesized that people would not take their children to local parks regardless of a nature prescription unless there were significant changes to enhance safety in these environments. She emphasized, “…you have to create (structured) activities and you have to do it at a safe time and you have to do it in a safe way because nobody wants to bring their children out anywhere and have to duck bullets.” Another mother suggested that a nature prescription would have to be accompanied by resources to travel to outdoor green spaces beyond the local neighborhood.” I would probably say you need to Uber me somewhere,” she explained, due to both safety concerns and not wanting to use her financial resources (gas) to drive to a park.

## 4. Discussion

While our participants acknowledged physical and mental benefits of being outdoors, in nature and green space, many spoke about unsafe and unmaintained conditions of their local parks, which were unwelcoming to families and children and deterred use. Local parks were described as missing desired features, such as gates for children’s safety, police surveillance, and structured activities. Some participants traveled outside their neighborhoods of residence to engage in exposure to natural and green environments, but described the financial resources and motivation required to do so. A number of participants also discussed how the poor upkeep of their local parks reflected an unequal distribution of safe and desirable green spaces across the city landscape, comparing their local parks to more well-maintained parks in predominantly white and higher-resource neighborhoods. 

Insights from focus group interviews highlighted important social and environmental considerations that need to be integrated as part of planning and maintaining nature prescriptions as a clinical health intervention. Programs, particularly in urban areas, may need to build additional supports that enable patients and their families to overcome individual and environmental barriers to nature-prescription adherence. Beyond educating patients and their families about local outdoor resources, more support may be required to generate a more normative community practice of spending time in neighborhood parks. This may require provision of programs or guided activities, and incentives. The impact of offering these kinds of supports has been shown to be effective at ensuring adherence and improving health in longitudinal cohort studies [17,61,62]. These findings align with those of a previous study of resident perceptions of parks to inform a new park prescription program [54].

Targeted improvements to neighborhood parks might also increase accessibility and adherence. In Philadelphia, PA there is strong evidence that enhancing the quantity and quality of green spaces can improve population health. A randomized controlled trial of cleaning-and-greening vacant lots demonstrated that after the intervention, crime around these lots was reduced and nearby residents not only increased their use of these outdoor spaces for social and recreational activities but also experienced improved perceptions of their mental health [49]. Beyond improving quality and access to local parks, it may also be necessary to provide transportation to parks outside of low-resource city neighborhoods. 

Our findings have several limitations. The sample has limited diversity, since participants were all native English speakers from four relatively low-resourced neighborhoods within a large city. Though the findings of this research are informed by the environmental and social context of specific residents and neighborhoods in Philadelphia, they may be applicable to similar urban neighborhoods and communities. Participants represent a convenience sample of people willing to talk about nature and health. This self-selected group may have been motivated to articulate viewpoints based on their personal interest in specific local environments. Furthermore, there were differences between focus group locations in terms of local demographic background and proximity and density of nearby parks. While our sample size did not allow us to make comparisons between study locations, future research could explore whether these factors play a role in experiences, knowledge, and perceptions, and thereby implications for practice.

This study offers new descriptive insights and a range of contextual concerns that should be considered for current and future nature prescription interventions targeted toward children and their caregivers in urban environments. Further experimental evaluation, via partnerships between physicians, health care systems, government and non-governmental agencies, and residents, can uncover the effect of individual and environmental approaches on patients’ ability to spend more time outdoors.

## 5. Conclusions

Despite knowledge of the location and features of local parks in Philadelphia, community members had mixed perceptions of the benefits and deterrent to use of these spaces. Encouraging green space exposure for health promotion may not be as simple as offering information to guide people to city parks (both local and more remote); ways to reduce barriers related to financial resources, physical health, and public safety may also need to be incorporated. 

## Figures and Tables

**Table 1:** Participant characteristics.

Demographics	*N* (%)
Gender	
Female	30 (71.4%)
Male	9 (21.4%)
Missing	3 (7.1%)
Age	
18–24	4 (9.5%)
25–34	5 (12%)
35–44	11 (26.2%)
45–54	9 (21.4%)
55–64	6 (14.2%)
65–74	2 (4.8%)
Missing	5 (12%)
Race (self-defined)	
Black	31 (73.8%)
Biracial	1 (2.4%)
White	1 (2.4%)
Mediterranean/MidEastern	1 (2.4%)
Puerto Rican	1 (2.4%)
Missing	7 (16.7%)

**Table 2:** Neighborhood characteristics at each focus group location.

Neighborhood Characteristics	Focus Group Location			
	South Philadelphia	West Philadelphia Site 1	West Philadelphia Site 2	Germantown
Racial makeup, %				
White	36	1	4	7
Black/African American	19	98	91	92
Asian	43	0	0	1
Family Households, %				
Male Householder, No Wife Present	8	12	12	8
Female Householder, No Husband Present	17	25	32	25
Median Household Income (In 2016 Inflation Adjusted Dollars)	$42,903	$26,814	$20,840	$31,299
Renter Occupied Housing Units, %	43	52	74	52
Population Living Below Poverty, %	23	28	41	34

**Table 3:** How participants access information about local outdoor green spaces.

Category	Resource
• Internet resources	• MeetUp.com; • Social Media outlets; • Facebook; • Facebook groups; • Twitter; • LinkedIn; • Township websites; • Using Google.
• Postings in the community	• Fliers; • At the library; • At community centers.
• Advertisements	• Local TV stations; • Community newspapers.
• Personal networks	• Word-of-mouth.

**Table 4:** Barriers discouraging time spent in nature.

Barriers	Specific Concerns
Safety concerns	Fear of being robbed; General feelings of not being safe; Lack of police presence; People drinking alcohol; People using illegal drugs; Prostitution; Trash posing health risks (e.g., drug paraphernalia such as syringes); Violence and crime in the community (e.g., rapes, stabbings, and shootings); Unrestrained dogs.
Dislike of things associated with nature environments	Bugs; Grass; Pollution; Risk of sunburn; Wild animals.
Financial hardship	Lack of access and funds to travel to desirable outdoor spaces.
Medical conditions affecting physical function	Flair up of chronic conditions; Not feeling well.
Outdoor conditions	Bad weather; Conditions created negative health experiences (e.g., allergies, asthma).
Physical conditions of spaces	Lack of seating; Presence of trash (e.g., dumping of household items like mattresses); Poor condition/not cared for park area.
